# Effects of Melatonin Supplementation on the Aortic Wall in a Diet-Induced Obesity Rat Model

**DOI:** 10.7759/cureus.33333

**Published:** 2023-01-03

**Authors:** Dariya Chivchibashi-Pavlova, George S Stoyanov, Kameliya Bratoeva

**Affiliations:** 1 Physiology and Pathophysiology, Medical University of Varna, Varna, BGR; 2 General and Clinical Pathology, St. Marina University Hospital, Varna, BGR; 3 General and Clinical Pathology/Forensic Medicine and Deontology, Medical University of Varna, Varna, BGR

**Keywords:** animal model, obesity, fructose, melatonin, aorta

## Abstract

Background

Cardiovascular disease (CVD) is still the leading cause of death globally. Alterations in the arterial wall architecture predict CVD morbidity and mortality and are associated with other CVD risk factors. Aortic wall thickness is closely linked to short- and long-term CVD morbidity and mortality, even without pronounced atherosclerotic changes. Obesity increases the risk of a broad spectrum of pathologies with vascular manifestation, which are often pathogenically associated with chronic oxidative stress and inflammatory response. Hence, as an antioxidant and anti-inflammatory agent, the pineal gland hormone melatonin is expected to have vasoprotective effects. This study evaluated the effects of melatonin supplementation on aortic wall thickness by assessing the cross-sectional associations of abdominal obesity with aortic intima-media thickness in a diet-induced obesity rat model.

Methodology

The model comprised of male Wistar rats that were on a high-fructose diet (HFD) (20% glucose-fructose corn syrup) for 12 weeks; the rats were divided into four groups (n = 8): control, HFD, HFD and melatonin supplementation (*per os* - 4 mg/kg/24h), and control and melatonin supplementation. All rats received a standard rodent diet and tap water. Zoometric measurements and the Lee index were calculated. Morphometric analysis of the abdominal aorta was performed by staining with hematoxylin-eosin and measuring the thickness of the abdominal aortic wall. For this, we used the Aperio Image Scope software. To evaluate the functional properties of the abdominal aorta, the modified Kernogan’s index (KI) was employed.

Results

The results showed significantly elevated body weight (Lee index), KI, and wall thickness of the *aorta abdominalis* with morphometric changes in the vessel wall in HFD rats compared to the control group. Melatonin supplementation prevented these changes.

Conclusions

The administration of HFD to Wistar rats led to pathomorphological and morphometric changes in their abdominal aorta, which constitute the main diagnostic criteria of endothelial dysfunction. Melatonin supplementation regressed vascular wall remodeling and restored its functional capacity.

## Introduction

Cardiovascular disease (CVD) is still the leading cause of death globally [1]. Alterations in the arterial wall architecture predict CVD morbidity and mortality and are associated with other CVD risk factors. Aortic wall thickness is closely linked to short- and long-term CVD morbidity and mortality [2,3], even without pronounced atherosclerotic changes [4,5].

Obesity increases the risk of a broad spectrum of pathologies with vascular manifestations, which are most commonly pathogenically associated with chronic oxidative stress and inflammatory response. Furthermore, obesity is considered an independent risk factor for CVD, as it directly affects hemodynamics and is related to endothelial dysfunction (ED), which is an indicator of multiple cardiovascular and metabolic disorders [6-9].

ED precedes the development of irreversible changes in the vascular wall, which is typical for clinically manifested CVD. ED is commonly characterized at later stages by vascular smooth muscle cell (VSMC) proliferation and thickening of the intima-media complex of the vascular wall [10,11]. In the case of obesity, these changes follow the development of chronic low-grade inflammation and oxidative stress due to adipose tissue dysfunction [9,12,13]. Expansion of visceral adipose tissue is linked to inflammatory infiltration, and these events are an essential contributor to systemic inflammation. A clear explanation for the accumulation of immune cells in adipose tissue is uncertain; one potential contributing factor is oxidative stress. Obesity is usually linked to elevated reactive oxygen or nitrogen levels and impaired antioxidant defenses [14].

According to the notion that obesity leads to vascular disorders due to chronic low-grade inflammation and oxidative stress, the pineal gland hormone melatonin, an antioxidant and anti-inflammatory agent, is expected to have vasoprotective effects. Indeed, according to various studies, melatonin supplementation might prevent end-organ damage in oxidative stress-related pathologies such as hypertension, atherosclerosis, cancer, and others [15-17]. It has been demonstrated that melatonin and its metabolites search for various free radicals in body fluids and cells in both in vitro and in vivo studies [8,18-20]. In addition, melatonin plays a crucial role in activating antioxidant defenses such as superoxide dismutase, catalase, glutathione peroxidase, glutathione reductase, and glucose-6-phosphate dehydrogenase [8]. 

In this study, we evaluate the effect of melatonin supplementation on the aortic wall in a diet-induced obesity rat model. Specifically, we assess the cross-sectional associations between abdominal obesity and aortic intima-media thickness.

## Materials and methods

Animals and treatments

This study was performed on 32 male Wistar rats. The rats were housed in specialized cages for 12 weeks in a well-ventilated room with a light/dark cycle of 12:12 (in hours) at 21-23°C. The rats were fed with standard rat chow and tap water ad libitum. The experiment was approved by the Ethics Committee for Animal Experiments of Bulgaria (protocol number 272, 2019)

The rats were randomly divided into four groups (n = 8): (a) control group (C)-rats received standard rodent diet and tap water-(b) melatonin group (C+M)-rats received standard rodent diet and tap water and were administered melatonin “*per os*” (4 mg/kg/24h)-(c) fructose group (Fru; high-fructose diet (HFD))-rats received standard rodent diet and tap water supplemented with 20% fructose-and (d) fructose plus melatonin group (Fru+M)-rats received standard rodent diet and tap water supplemented with 20% fructose and were administered melatonin “*per os*” (4mg/kg/24h). At the end of the experimental study, all the rats were euthanized using ketamine anesthesia, and then, zoometric measurements were taken and their aorta was quickly removed.

Measurement of Lee obesity index

At the end of the experimental study, the rats were weighed while they were under ketamine anesthesia, and the naso-anal length (NAL), the length from the nose to the anus, was measured. The Lee index was calculated according to the following formula:

$Lee Index = \frac{\sqrt[(3)]{Weight}(g)}{NAL(mm)} x 10$

Morphometric analysis of the abdominal aorta

The aorta tissue samples were fixed in 4% paraformaldehyde for 48 hours and routinely embedded in paraffin. The samples were sliced with a thickness of 4μm into serial transversal sections, which were then mounted on slides and stained with hematoxylin-eosin (HE). We used the Aperio Image Scope software (v12.3.3.5048) to measure the thickness of the abdominal aortic wall and the thickness of the tunica intima-tunica media layers.

A modified Kernogan’s index (KI) was applied to evaluate the abdominal aorta's functional properties. The KI was calculated according to the following formula:

$Kernogan index = \frac{t.intima-t.media thickness (\mu m)}{inner aortic diameter(\mu m)}$

Statistical analysis

All results were expressed as means ± standard error of the mean (SEM) in the table. The statistical significance of the studied parameters was determined using Student's t-test. P-values less than 0.05 were regarded as significant. All tests were two-tailed. All statistical analyses were carried out using Graph Pad Prism 7.0 (GraphPad Software, San Diego, California, United States of America).

## Results

**Figure 1:**
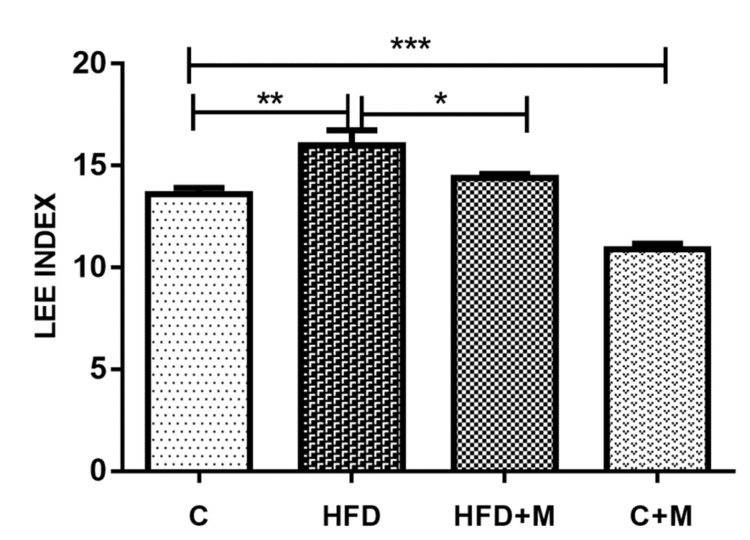
Lee obesity index calculated for all experimental animals. Mean levels ± standard error of the mean; n = 8; C: control group rats; HFD: rats on high-fructose diet; HFD+M: rats on high-fructose diet treated with melatonin; C+M: control group rats treated with melatonin. *p < 0.05; **p < 0.001; ***p < 0.0005, t-test.

The findings of this experimental study indicate that an HFD induced obesity in the experimental model. Lee index is considered an easy and precise method of evaluating obesity in experimental animals. Our results demonstrate an increased Lee index in rats in the HFD group compared to those in the control group (р < 0.001). On the other hand, melatonin supplementation in rats on HFD led to a statistically significant reduction in the Lee index at р < 0.05. Interestingly, the Lee index of rats in the C+M experimental group was also lower than those in the control group (р < 0.0005) (Figure 1).

**Figure 2:**
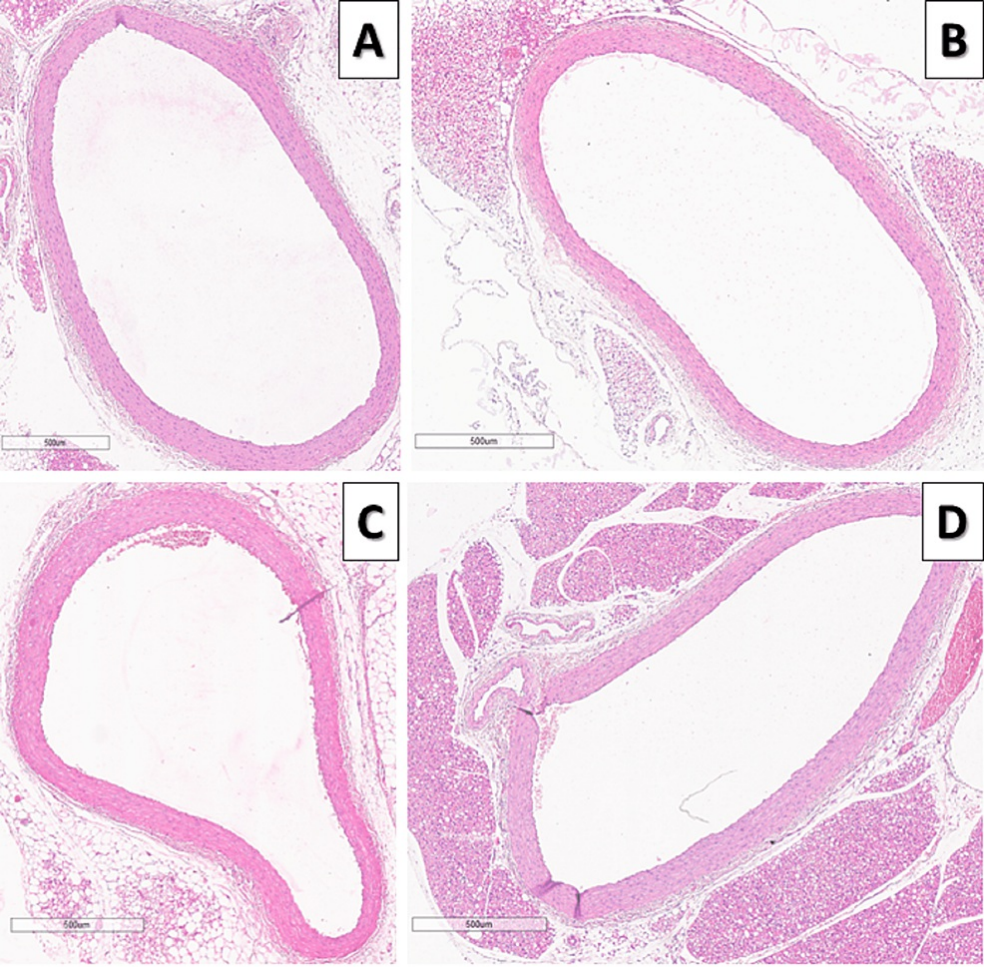
Effects of melatonin on aortic wall thickness. Representative microscopic photographs of HE-stained sections of the abdominal aorta in all experimental groups (magnification ×4) A: control group rats; B: control group rats treated with melatonin; C: rats on high-fructose diet; D: rats on high-fructose diet treated with melatonin; HE: heatoxylin and eosin.

**Figure 3:**
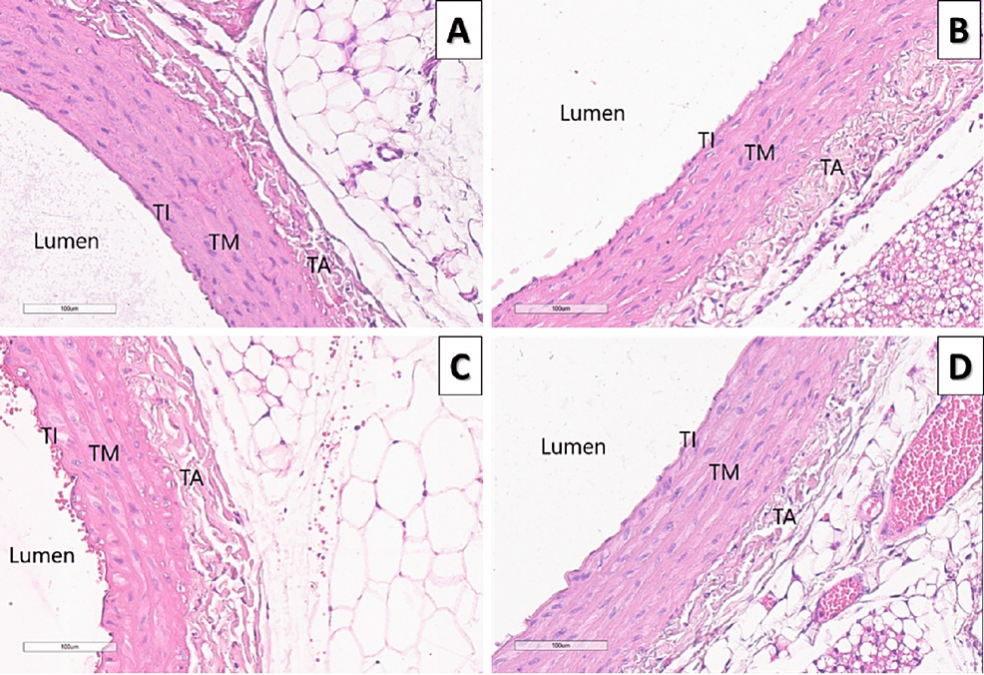
Effects of melatonin on aortic wall thickness. Representative microscopic photographs of HE-stained sections of the abdominal aorta in all experimental groups (magnification ×40) A: control group rats; B: control group rats treated with melatonin; C: rats on high-fructose diet; D: rats on high-fructose diet treated with melatonin; TI: *tunica intima*; TM: *tunica media*; TA: *tunica adventicia*; HE: heatoxylin and eosin.

Results from the routine histological and morphometric analysis of aorta abdominalis demonstrate increased aortic wall thickness (р < 0.001), tunica intima-tunica media complex thickening (р < 0.001), and inner lumen narrowing (р < 0.05) in HFD rats compared to the control group. Meanwhile, rats on HFD and melatonin supplementation demonstrate a reduction in aortic wall thickness and thickness of the tunica intima-tunica media complex compared to the HFD group, and the size of the inner lumen was similar to that of rats in the control group (р < 0.001) (Figure 2, Figure 3, and Table 1).

**Table 1:** Calculation of the average thickness of tunica intima-tunica media in the abdominal aortic wall by the Aperio Image Scope software (v12.3.3.5048), (Leica Biosystems Inc., Illinois, United States of America) Mean levels ± standard error of the mean; n = 8; C: control group rats; HFD: fructose-drinking rats; HFD+M: fructose-drinking rats on melatonin supplementation; C+M: control group rats treated with melatonin. а р < 0.05: statistical significance between HFD and C; ааа р < 0.001: statistical significance between HFD and C; *р < 0.01: statistical significance between HFD and HFD+M; ***р < 0.001: statistical significance between HFD+M and HFD, t-test.

Morphometric indicator for different groups	C	HFD	HFD+M	C+M
Entire aortic wall thickness (μм)	119.9 ± 1.42	152.2 ± 3.09^ааа^	133.0 ± 1.32***	118.7 ± 1.38
Aortic wall thickness- *tunica intima-tunica media* complex (μм)	88.81 ± 0.78	113.7 ± 1.07^ааа^	105.0± 0.63***	87.93± 0.99
Inner diameter (μм)	1.627± 0.04	1.425± 0.07^а^	1.816± 0.03***	1.700± 0.05

**Figure 4:**
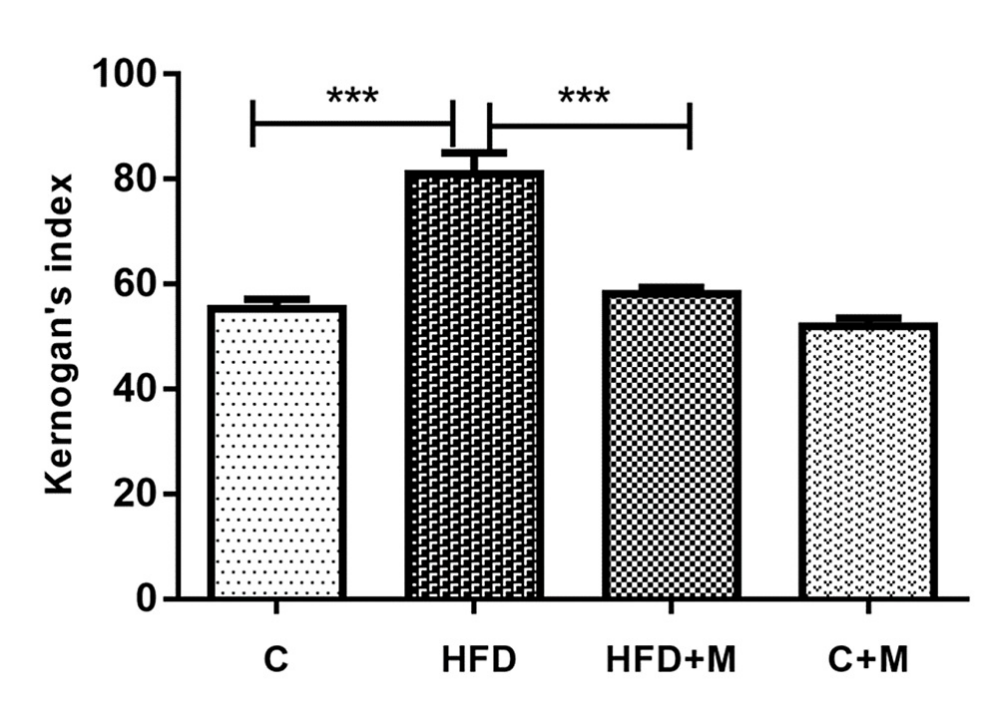
Kernogan's index for all experimental groups. Mean ± standard error of the mean; n = 8; C: control group rats; HFD: fructose-drinking rats; HFD+M: fructose-drinking rats treated with melatonin; C+M: control group rats treated with melatonin. ***p< 0.0001, t-test

In this experimental study, to analyze the functional capacity of the aorta abdominalis, KI was applied. According to our observations, rats in the HFD group demonstrate higher KI than the control group (р < 0.0001). However, rats on HFD and melatonin supplementation present a statistically significant reduction in the KI value compared to the HFD group (р < 0.0001). Both experimental groups on melatonin supplementation showed a KI similar to the control group (Figure 4).

## Discussion

The results of this experimental study indicate that HFD consumption for 12 weeks leads to a visceral type of obesity (increased Lee index values). Pathogenesis involved in the development of fructose-induced obesity includes the following potential mechanisms: 1) leptin resistance, 2) true hyperphagia, 3) positive energy balance, and 4) increased body weight [21,22]. Our results also show that melatonin supplementation statistically significantly reduces the Lee index value in HFD rats treated with melatonin compared to those in the HFD group. Furthermore, melatonin supplementation affects the Lee index value of rats' traditional diet. Currently, mechanisms involved in the therapeutic effect of melatonin are poorly understood. Most probably, melatonin supplementation affects the process of lipogenesis and reduces body weight even in healthy experimental animals (C+M). Multiple empirical studies have reported on the pathogenetic correlation with obesity, and it was reported that risks for ED development, and hence, cardiovascular pathology, are higher in the case of weight gain and increased abdominal circumference [21,23-25]. It is believed that HFD induces oxidative stress and low-grade inflammation in multiple tissues and organs, including vasculature. Reactive oxygen species reduce nitrous (NO) bioavailability [26,27]. The inhibited activity of endothelial nitric oxide synthase and/or accelerated NO decay, results in impaired endothelium-dependent vasodilation, increased risks for thrombotic events and pathological remodeling of the vascular wall associated with ED [28]. According to Pektas et al.’s [29] experimental study on rats, a fructose diet is tightly linked to impaired endothelium-dependent vasodilation and reduced NO bioavailability in the aorta. We may hypothesize that HFD induces oxidative stress and reduces NO bioavailability in rats and this is most probably due to the early pathomorphological changes in aorta abdominalis-remodeling and impaired functional capacity. This explains the statistically significant thickening of the aortic wall, tunica intima-tunica media complex, and the reduction in the inner lumen size of rats in the HFD experimental group compared to those in the control group.

Furthermore, the results show a statistically higher KI value for the HFD group compared to the control group. This observation demonstrates the effect of HFD on both the morphological alterations in the aortic wall and the functional capacity of the vessel. Melatonin supplementation drastically reduces aortic wall thickness and thickness of the tunica intima-tunica media complex and improves the inner diameter changes in the HFD experimental group. Also, melatonin supplementation (statistically significantly) improves the KI of rats on HFD.

Mechanisms involved in the vasculoprotective effects of melatonin supplementation in the context of fructose-induced obesity are poorly understood. Melatonin probably improves metabolic alterations and adipose tissue dysfunction, leading to obesity via its antioxidant and anti-inflammatory nature. Hence, we may hypothesize that melatonin has endothelial protective potential.

Study limitations and future perspectives

Although this study focuses on the effects of melatonin on aortic morphometry in an obesity rat model, multiple other facets may be addressed in the future. Future research can consider animal obesity models induced with lipids to examine the effects of melatonin on lipid metabolism, which was not the focus of this study. Mor, melatonin's effects on other CVD factors such as blood pressure, serum glucose levels, and cardiac rhythm can be studied. Furthermore, especially with regard to CVD, morphology alone sometimes is not sufficient for determining the outcome, since vascular and cardiac compliance and elasticity should also be considered.

Unlike other experimental models, the data transition from bench to bedside is relatively easy for this study, as melatonin is an over-the-counter supplement used primarily for its effect on the sleep cycle. Hence, a cohort of healthy CVD patients supplementing with melatonin can easily be studied clinically for melatonin’s clinical benefits on CVD, risk factors, and prevention of those risk factors. Furthermore, such a detailed clinical study would better help understand most clinical effects, including laboratory constellations, associated with melatonin supplementation and also identify the possible side effects, which cannot be concluded from implementing an animal model alone.

## Conclusions

This experimental study demonstrated that melatonin supplementation drastically reduces aortic wall thickness and tunica intima-tunica media complex thickness and improves inner diameter size changes and the vessel's functional capacity in rats having fructose-induced obesity. Our results demonstrate that melatonin supplementation has the potential of a promising therapeutic option in vascular pathology linked to the visceral type of obesity.
